# Stability evaluation of rock pillar between twin tunnels using the YAI

**DOI:** 10.1038/s41598-023-40167-9

**Published:** 2023-08-14

**Authors:** Huijian Zhang, Gongning Liu, Weixiong Liu, Zekun Chen, Zengrun Miao, Qiuyang Liu

**Affiliations:** 1 Key Laboratory of Transportation Tunnel Engineering, Ministry of Education, School of Civil Engineering, Southwest Jiaotong University, Chengdu, 610031 China; 2 China Railway Construction Bridge Engineering Bureau Group Co., Ltd., Tianjin, 300300 China

**Keywords:** Civil engineering, Mechanical engineering

## Abstract

The stability of rock pillar is crucial for ensuring the construction safety of twin tunnels with small clearance, especially when transitioning from the traditional left–right tunnel layouts to the up-down configurations due to complex and variable site constraints. However, there are limited researches on the evaluation and comparative study of the stability of these two types of rock pillars in twin tunnels. This paper introduces the yield approach index (YAI) as a measure to assess the stability of rock pillar in twin tunnels with small clearance, and various influencing factors including side pressure coefficient (SPC), stress release rate (SRR), and the thickness of rock pillar (characterised by the ratio of rock pillar thickness to tunnel diameter, RPT/TD) are considered in the analysis. The study compares and analyzes the stability differences of the rock pillar in different situations. It is observed that the two sides of up-down tunnels pose a higher risk while the rock pillar in the left–right configuration being the most vulnerable. The stability of the rock pillar between the up-down tunnels is significantly higher than that of the left–right tunnels under similar conditions. Moreover, the up-down tunnels exhibit greater sensitivity to SPC, whereas the left–right tunnels are more sensitive to SRR. Additionally, the study reveals that increasing the RPT/TD can effectively improve the stability of the rock pillar within a specific range (1/4 to 2/3). The research method and obtained results of this paper can provide some important references for the stability evaluation and design of twin tunnels with small clearance.

## Introduction

**Figure 1:**
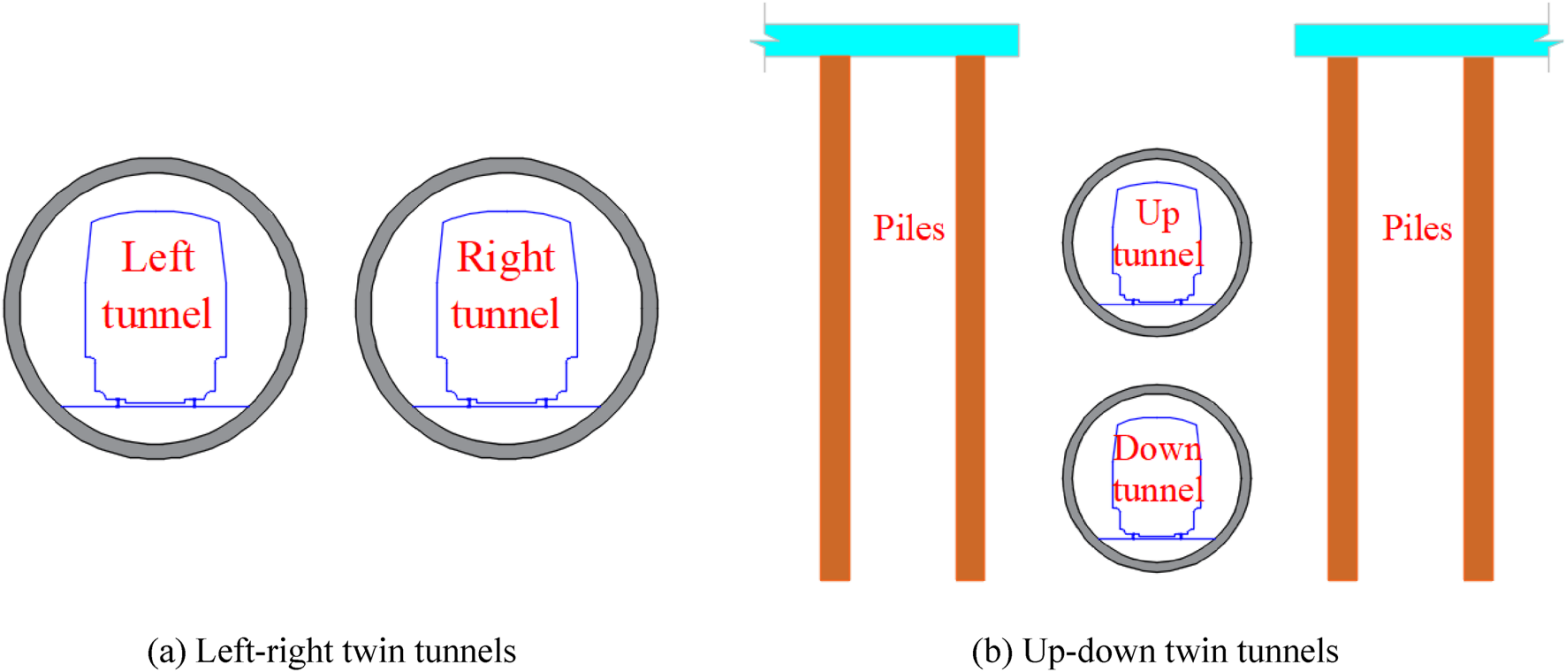
Common layouts of twin tunnels with a small clearance.

In practical engineering, tunnel stability has always been the focus of scholars^1–6^. In metro engineering, twin tunnels are commonly designed as left–right tunnels at the same elevation, as shown in Fig. 1a. However, in some cases, to avoid the existing piles or any other structures, the up-down tunnels are also adopted, as shown in Fig. 1b.

Generally, the distance between the twin tunnels is variable in different projects or sections within one project. If the twin tunnels are far enough from each other, each tunnel can be analyzed separately without considering the influence of another tunnel. However, if the two tunnels come closer (i.e. the clearance between twin tunnels is less than the code requirement^7^), the stress state of rock pillar between two tunnels will be significantly affected. The influence is usually decided by the ground properties, in-situ stress, tunnel span, rock pillar thickness, and excavation methods. Existing research concentrates on the interaction between left–right twin tunnels^8–13^, mechanical characteristics during tunnel construction^14–16^, ground settlement characteristic^17,18^, optimization of rock pillar thickness and construction sequences^19–23^, the prediction method of tunnel convergence^24^, as well as the influence of twin tunnels on the surrounding buildings^25^. Previous studies have also showed that excavation methods have a significant influence on the stability of twin tunnels^26,27^. Additionally, studies have examined the construction stability of up-down tunnels, primarily considering factors such as tunnel oblique angle, lining method, dismantling sequence^28–33^. On the other hand, scholars have conducted certain research on the stability of the overall two tunnels and proposed corresponding evaluation methods and suggested measures for strengthening^34^. Jiang et al*.*^35^ demonstrated the whole failure process of two tunnels using numerical simulation based on a 3D printed sandstone analog model and proposed the estimating method of safety factor for the overall stability of twin tunnels. The results showed that the connectivity of the plastic strain could be the conservative instability criterion, while the turning point of tunnel deformation could be the overall failure criterion of twin tunnels. Based on the limit equilibrium study of many failure patterns, Lyu et al*.*^36^ calculated the surrounding rock pressure of two tunnels and verified it through numerical analysis and field data. It was found that the vertical pressure was greatly affected by the soil thickness and the properties about the surrounding soil. Fan et al*.*^37^ studied the stress redistribution and displacement characteristics of adjacent tunnels led by the excavation of bilateral deep foundation pits and proposed corresponding reinforcement measures.

According to the previous literature, there has been a certain amount of research on twin tunnels with small clearance, and most of them are about the construction mechanics of the overall tunnel, optimization of rock pillar thickness and construction sequences, the reinforcement method, and effect of rock pillar, while the comparative study on the mechanical stability of two tunnels with small clearance and different layouts (left–right and up-down) is rarely involved. The stability of rock pillar of twin tunnels with small clearance is vital in engineering design and construction. Due to the disturbing construction, when the layout of the two tunnels is diverse, the mechanical behavior of the rock pillar is also different. During the construction process, which may cause a large degree of damage and deterioration in the rock pillar of twin tunnels, resulting in a poor safety state of rock pillar, it is easy to cause problems such as instability of surrounding rock and tunnel collapse. Therefore, the stability of rock pillar has important practical significance in tunnel construction and even normal operation in the later period.

This paper aims to provide a systematic and straightforward analysis of the mechanical stability of twin tunnels with different layouts (i.e. left–right and up-down) through numerical simulation and theoretical analysis using YAI and Mohr’s circle. Firstly, the numerical simulation is calibrated by conducting theoretical analysis using YAI and Mohr’s circle. This step ensures the accuracy and reliability of the simulation results. Next, the mechanical stability of twin tunnels with different layouts is extensively analyzed. The focus is on evaluating the stability differences between the left–right and up-down twin tunnel configurations. Furthermore, a comparative analysis is performed to assess the influence of various parameters, including SPC, SRR, and RPT/TD, on the stability differences and change law of the rock pillar for both left–right and up-down twin tunnels. The findings of this study are presented, and corresponding suggestions are also provided based on the results obtained. The goal is to offer valuable references for similar engineering cases in the future, aiding in decision-making and improving the overall stability and safety of twin tunnel designs.

## Evaluation index for the stability of rock pillar about twin tunnels

**Figure 2:**
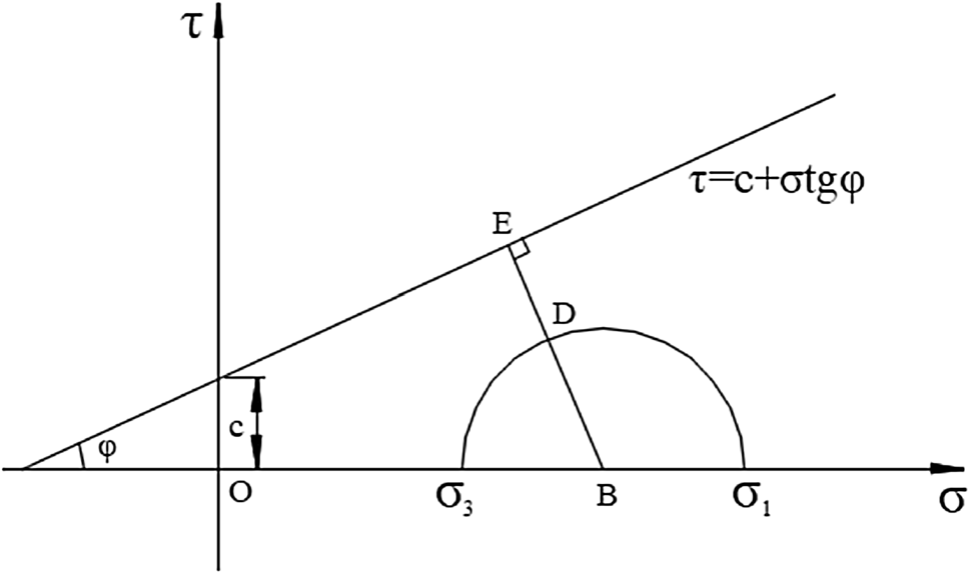
Sketch of the YAI using MC criterion.

The YAI index can quantitatively evaluate the extent that which the current state of material approaches the yield state^38^. Therefore, in this paper, YAI is introduced to evaluate the stability evolution during the excavation of two tunnels, and the function of YAI is shown in Fig. 2 based on the Mohr–Coulomb criterion (MC).

YAI is defined as Eq. (1), *EB* and *BD* are calculated through Eqs. (2), (3),

1 $$ YAI = \frac{ED}{{EB}} = \frac{EB - BD}{{EB}} = 1 - \frac{BD}{{EB}}, $$

2 $$ EB = \sin \varphi \times [c \times ctg\varphi + (\sigma_{1} + \sigma_{3} )/2] = c \times \cos \varphi + [(\sigma_{1} + \sigma_{3} )\sin \varphi ]/2, $$

3 $$ BD = \frac{{\sigma_{1} - \sigma_{3} }}{2}. $$

Then YAI can be abbreviated as Eq. (4)

4 $$YAI = 1 - \frac{{\sigma_{1} - \sigma_{3} }}{{2c\cos \varphi + (\sigma_{1} + \sigma_{3} )\sin \varphi }}.$$

The value of YAI is between 0 and 1. When YAI = 0, the point is on the yield surface, and the contrary, it is in a relatively safe state when YAI = 1. The rock will be safer with the increase of YAI. For the same material with a certain failure envelope, YAI is exclusively determined by the radius and center of the Mohr circle.

## Stability differences of rock pillar between left–right tunnels and up-down tunnels

### Numerical model and calculation parameter

**Figure 3:**
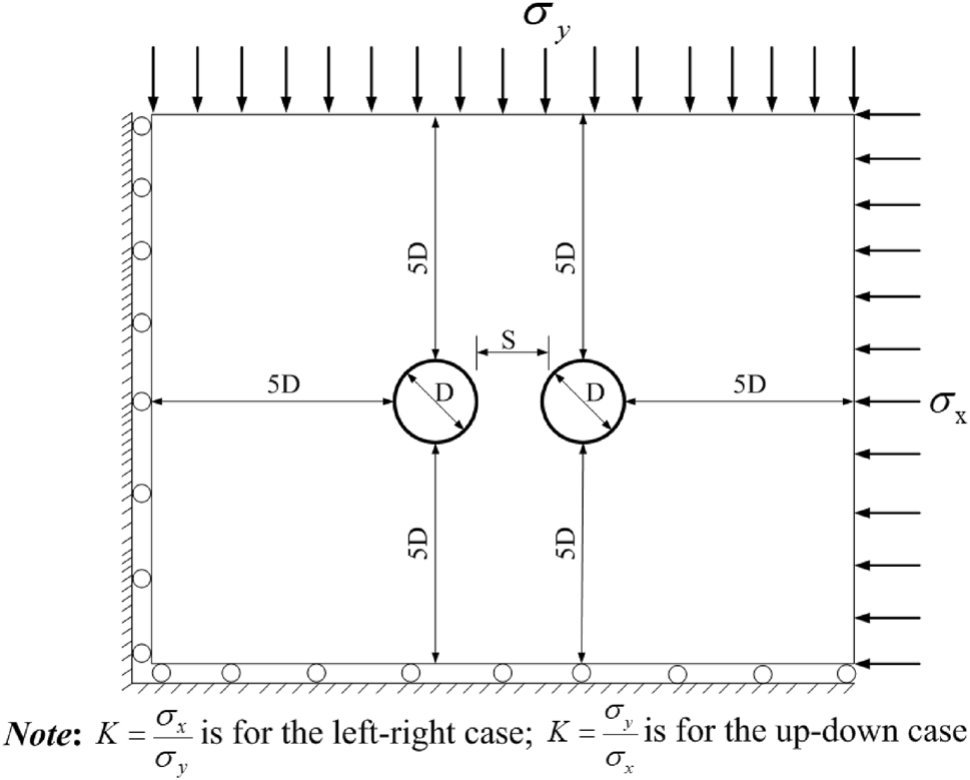
Theorical model for twin tunnels ($\sigma_{x}$ < $\sigma_{y}$ is for left–right case and $\sigma_{x}$ > $\sigma_{y}$ is for up-down case).

FLAC^3D^ software is adopted for numerical simulation. The calculation model including the twin tunnels and the boundary conditions^12^ can be found in Fig. 3.

**Figure 4:**
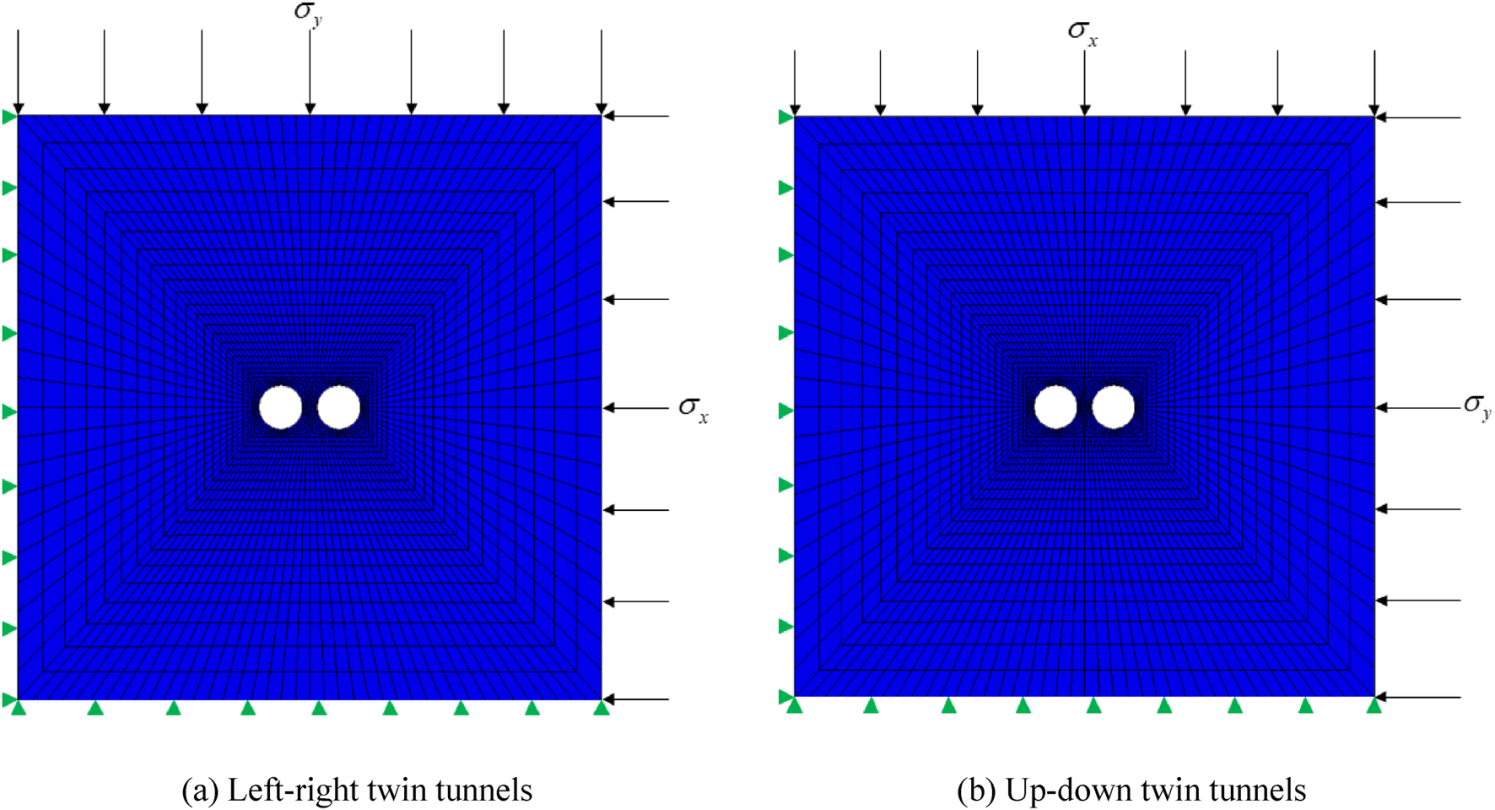
Numerical calculation model.

The diameters (*D*) of the twin tunnels are the same, and the outer boundaries are located at least five times the diameter (*5D*) to minimize the boundary effects^39,40^. The initial stress field is produced by applying $\sigma_{x}$ at the right boundary and $\sigma_{y}$ at the top boundary. Although it is illustrated as left–right twin tunnels in the Fig. 3, for the up-down twin tunnels case, just exchange $\sigma_{x}$ and $\sigma_{y}$ (only exchange values, keep the location of $\sigma_{x}$ and $\sigma_{y}$ unchanged) when compared with the left–right case. Accordingly, ${\text{K}} = \frac{{\sigma_{y} }}{{\sigma_{x} }}$ is for the up-down case while ${\text{K}} = \frac{{\sigma_{x} }}{{\sigma_{y} }}$ is for the left–right case (*K* is the side pressure coefficient, which is equivalent to SPC). Since the diameter of the metro is usually taken as 6 m, and an RPT and stress field (SPC) are assumed temporarily here, these parameters will be changed to reveal the law in the next section. In this subsection, the variables mentioned in Fig. 3 are shown in Table 1, and the rock mass properties are taken as follows: Elastic modulus *E* = 2GPa; Poisson ratio *μ* = 0.3; cohesion *c* = 1.2 MPa; friction angle *Φ* = 33°. The numerical calculation model is shown in Fig. 4.

**Table 1:** Calculation parameters of twin tunnels.

Name	Left–right twin tunnels	Up-down twin tunnels
TD	6 m	6 m
RPT/TD	2/6	2/6
Initial stress field for X orientation ($\sigma_{{\text{x}}}$)	0.4 MPa	1.0 MPa
Initial stress field for Y orientation ($\sigma_{{\text{y}}}$)	1.0 MPa	0.4 MPa

The vertical movement is allowed while normal displacements of these planes are restrained in this study. In the initial stress field, for the left–right case, excavate the left tunnel first; for up-down case, excavate the up tunnel first. Each tunnel is full-face excavated and all tunnels are without lining. The surrounding rock obeys the *MC* failure criterion, and the stress circles in the whole process of the failure envelop can be made. The relative position of the Mohr circle and the failure enveloping line can assess the safety state of the structure, so YAI can be used to evaluate the safety margin.

### Calibrating of numerical simulation

**Figure 5:**
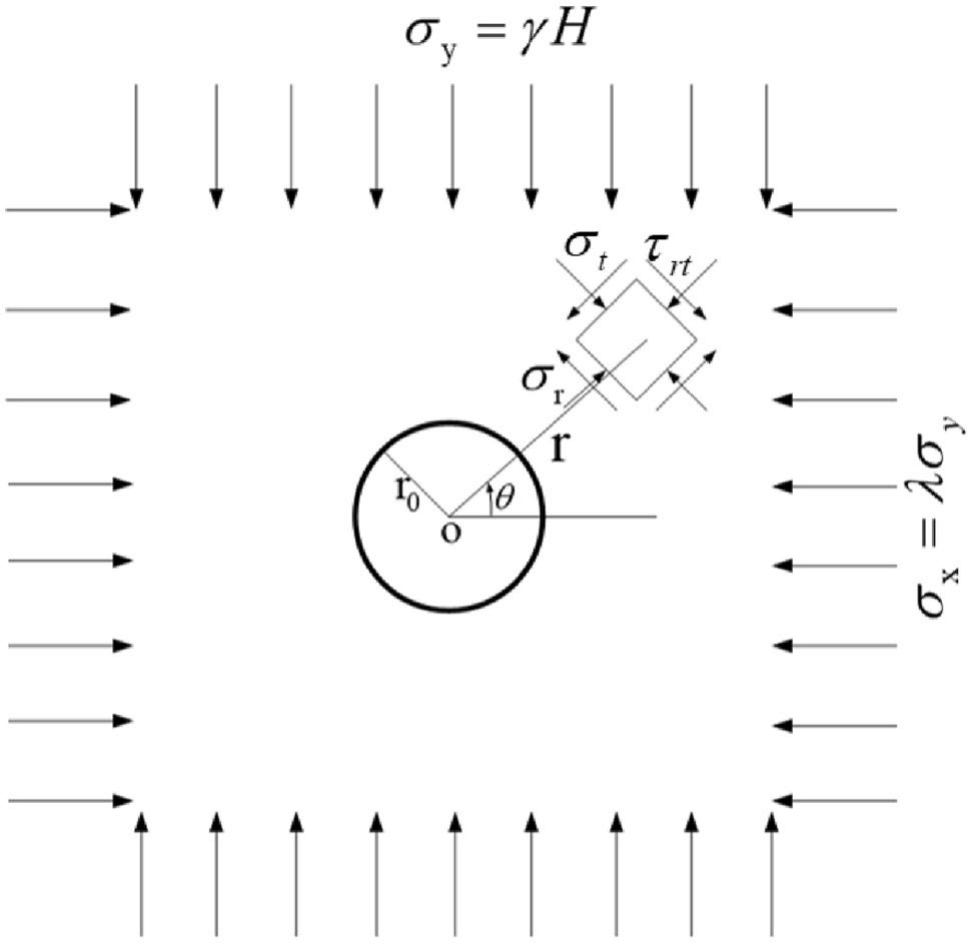
Theoretical calculation model.

To ensure the accuracy of the numerical calculation, it is necessary to compare them with the theoretical solution. The theoretical calculation model can be found in Fig. 5.

Theoretical solutions^41^ are expressed as Eqs. (5), (6), (7) and (8),

5 $$ \sigma_{r} = \frac{{\sigma_{z} }}{2}\left\lceil {(1 - \alpha^{2} )(1 + \lambda ) + (1 - 4\alpha^{2} + 3\alpha^{4} )(1 - \lambda )\cos 2\theta } \right\rceil , $$

6 $$\sigma_{t} = \frac{{\sigma_{z} }}{2}\left[ {(1 + \alpha^{2} )(1 + \lambda ) - (1 + 3\alpha^{4} )(1 - \lambda )\cos 2\theta } \right],$$

7 $$ \tau_{rt} = - \frac{{\sigma_{z} }}{2}(1 - \lambda )(1 + 2\alpha^{2} - 3\alpha^{4} )\sin 2\theta , $$

8 $$ \alpha = \frac{{{\text{r}}_{0} }}{r}, $$

where $\sigma_{r}$, $\sigma_{t}$ and $\tau_{rt}$ are the radial stress, tangential stress and shear stress at any point in the surrounding rock respectively, and the shear stress is positive when the normal outside the plane of action is consistent with the coordinate axis and the stress direction is opposite to the coordinate axis; $\lambda = \frac{{\sigma_{{\text{y}}} }}{{\sigma_{x} }}$ is equivalent to SPC; *r* and $\theta$ is the polar coordinate of any point in the surrounding rock;$\sigma_{z}$ is the initial ground stress; *r*_*0*_ is the tunnel radius.

The numerical results and theoretical results of $\sigma_{1}$ and $\sigma_{3}$ about a single tunnel under different SPC are obtained respectively, as presented in Table 2.

**Table 2:** Comparison between numerical results and theoretical results under different SPC.

SPC	Maximum principal stress ($\sigma_{1}$)/MPa		Relative error	Minimum principal stress ($\sigma_{3}$)/MPa		Relative error
	Theoretical value	Numerical value		Theoretical value	Numerical value	
0.2	1.717	1.728	0.64%	0.383	0.382	0.26%
0.4	1.679	1.688	0.54%	0.396	0.398	0.51%
0.6	1.640	1.648	0.49%	0.410	0.414	0.98%
0.8	1.601	1.609	0.50%	0.424	0.430	1.42%
1.0	1.563	1.570	0.45%	0.438	0.446	1.83%

Relative error = $$\frac{{\left| {{\text{Nu}}} \right.{\text{merical value}} - {\text{Theoretical val}}\left. {{\text{ue}}} \right|}}{{\text{Theoretical value}}}$$ × 100%.

In Table 2, the maximum principal stress decreases with the increase of SPC, while the minimum principal stress increases with the SPC. This data indicates that the numerical analysis results slightly exceed the theoretical values, although the disparity is negligible. Furthermore, this demonstrates a strong alignment between the numerical and theoretical analyses, affirming the validity of the numerical simulation. Hence, it can be concluded that the numerical method effectively captures the mechanical stability of an unlined tunnel. In the next section, the numerical simulation is used for the analysis of twin tunnels.

### Calculation results of left–right twin tunnels and up-down twin tunnels

In this section, the YAI and the distribution of the $\sigma_{1}$ of the left–right twin tunnels and the up-down twin tunnels are analyzed, and the distribution and range of the mechanical Mohr circle of these two layouts are also further analyzed, aiming to obtain their specific mechanical stability.

#### Stability analysis of left–right twin tunnels

**Figure 6:**
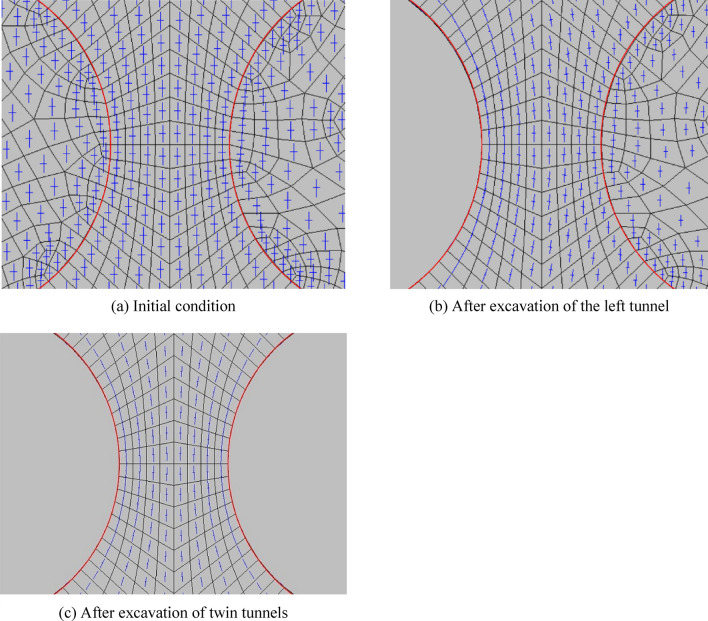
Distribution of the orientation of the $\sigma_{1}$ and $\sigma_{3}$.

**Figure 7:**
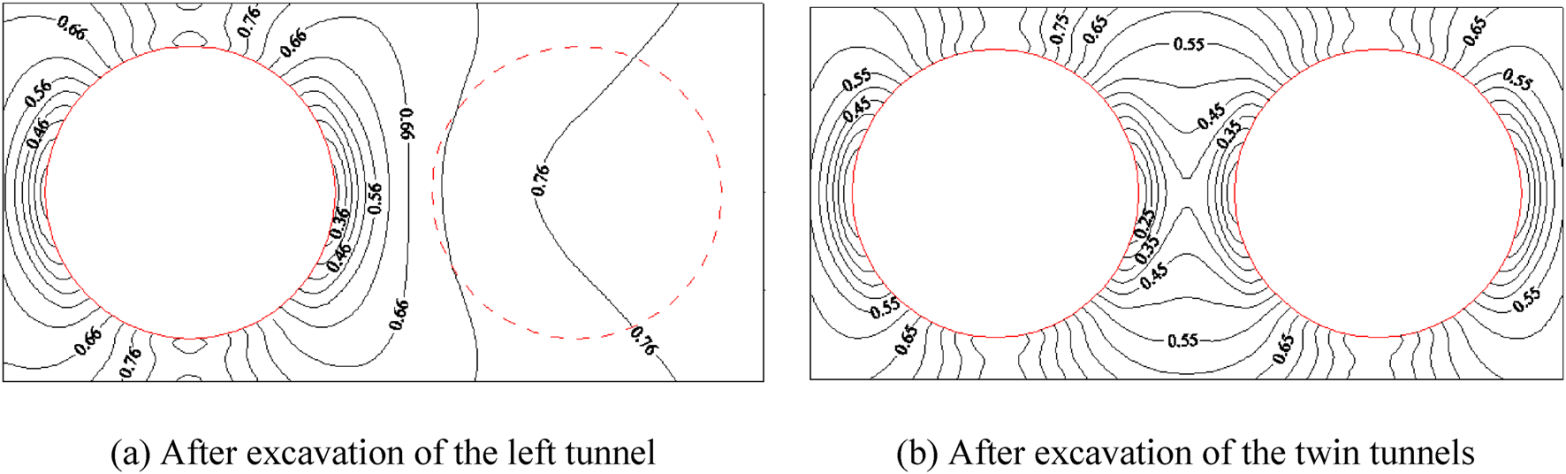
Distribution of YAI for the left–right twin tunnels during different excavation stages.

The calculation results of left–right twin tunnels are shown in Figs. 6, 7 (under the condition that SPC, SRR, and RPT/TD are 0.4, 100%, and 2/6, respectively).

Figure 6 illustrates the significant reduction in the principal stress of the tunnel’s surrounding area after the excavation of the left tunnel. This reduction can be observed by comparing the length of the blue line, which serves as a relative indicator of the stress value. The reduction is primarily characterized by a sharp decrease in the normal stress and a slower decrease in the tangential stress. Furthermore, the excavation of twin tunnels leads to a significant variation in the maximum principal stress, particularly in the normal stress, when compared to the initial state.

Figure 7 illustrates the relationship between the excavation of the left tunnel and the distribution of YAI. As the left tunnel is completely excavated, the density of YAI contours increases on both sides of the tunnel, with the highest density observed closer to the tunnel. Concurrently, the YAI value decreases from 0.76 to 0.36, indicating a decrease in safety. The numerical contour of YAI also exhibits a symmetrical distribution near the left tunnel, resembling an “ear-shaped” pattern on both the left and right sides. Upon excavating both tunnels, the YAI contours near the twin tunnels display symmetrical distribution. Notably, the rock pillar between the tunnels poses the greatest risk, as it exhibits the smallest YAI value of 0.25. Consequently, it is crucial to enhance the lining and monitoring of the rock pillar to prevent yield failure during the excavation of the left–right twin tunnels.

**Figure 8:**
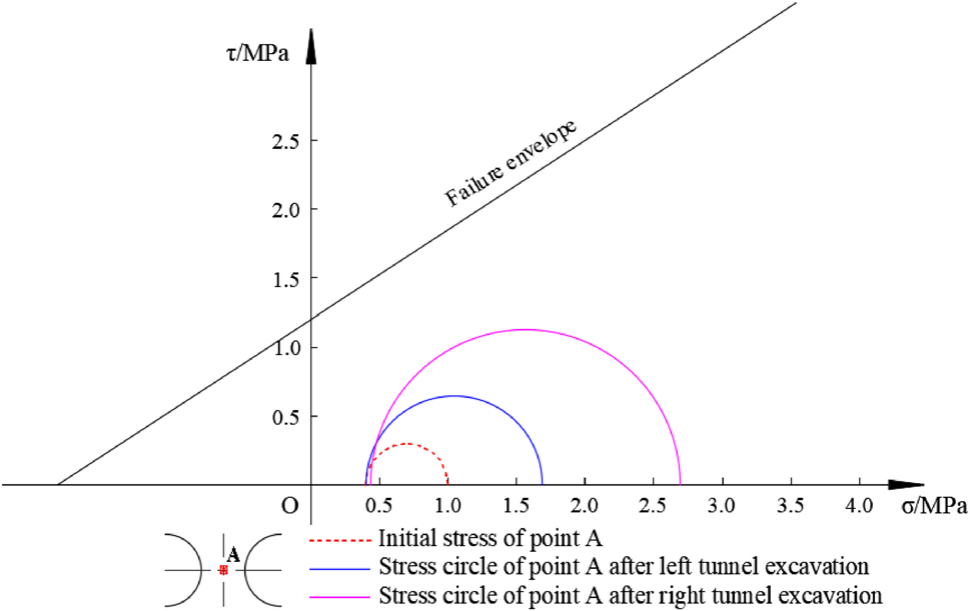
Mohr’s circle of left–right twin tunnels at point A.

Figure 8 displays the Mohr’s circle of the rock pillar (point A) based on the numerical simulation results of left–right twin tunnels.

Figure 8 illustrates the stress conditions in different situation. Initially, when the tunnel has not been excavated, the stress circle is small, indicating a safe state. However, upon excavation of one or two tunnels, the stress circle gradually expands and shifts towards the right direction. Although the stress circles remain within the failure envelope range, the excavation of two tunnels results in a significant enlargement of the stress circle, bringing it closer to the proximity of the failure envelope. This indicates that the rock pillar between the two tunnels is in a more unsafe state, making surrounding rock more susceptible to yielding.

#### Stability analysis of up-down twin tunnels

**Figure 9:**
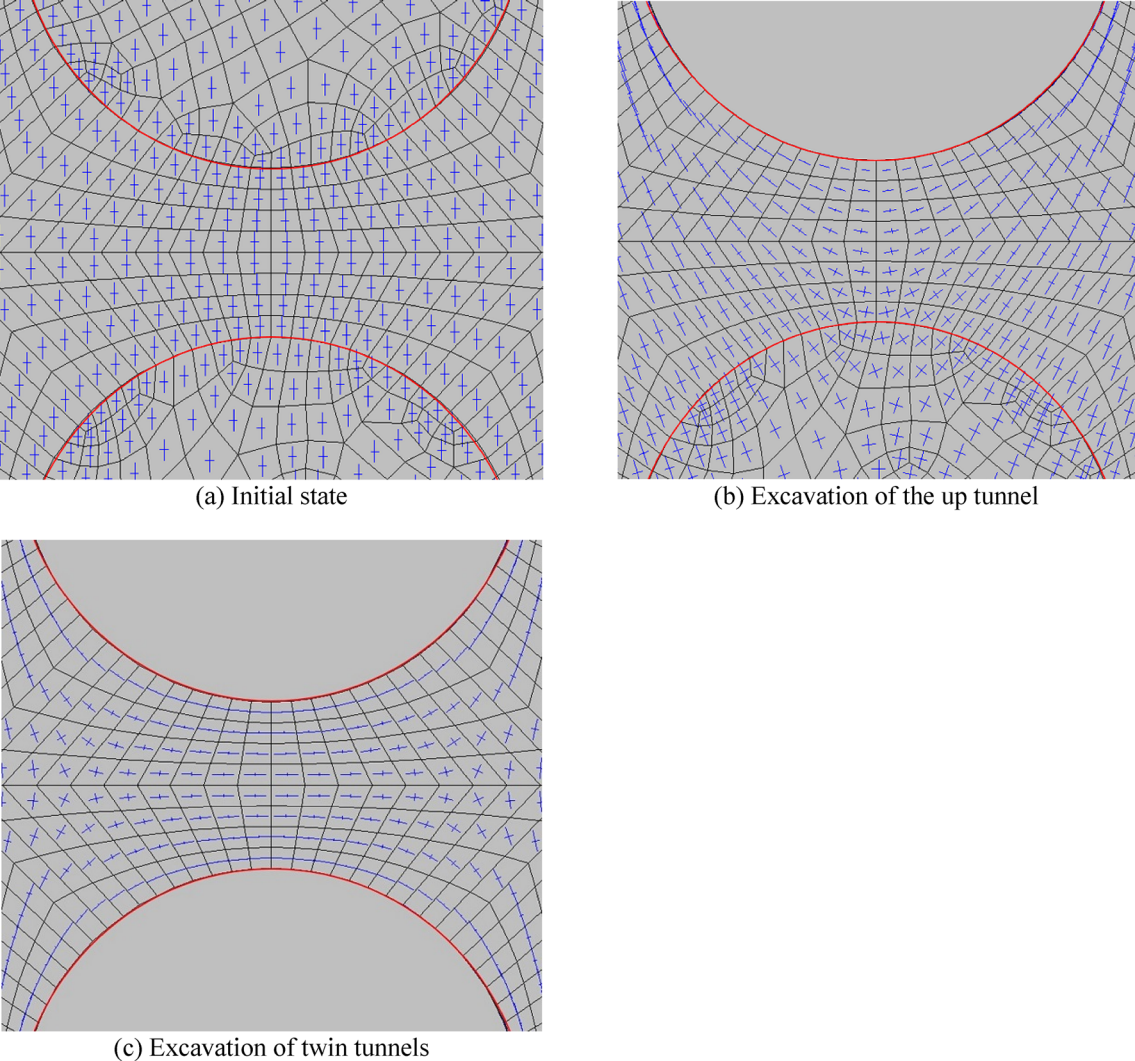
Distribution of the direction of $\sigma_{1}$ and $\sigma_{3}$.

**Figure 10:**
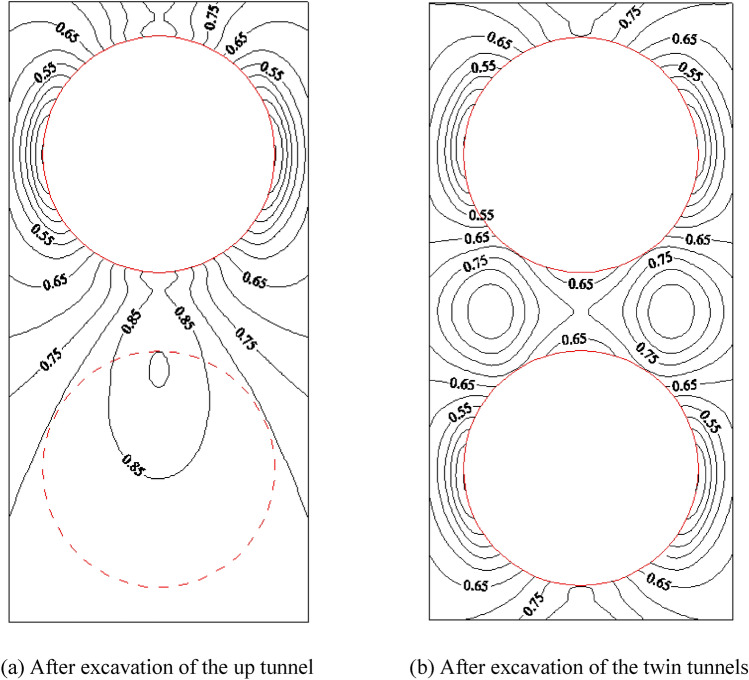
Distribution of YAI of up-down twin tunnels during different excavation stages.

Similarly, the calculation results of up-down twin tunnels are obtained through numerical simulation, as shown in Figs. 9, 10. Additionally, SPC, SRR, and RPT/TD are fixed at 0.4, 100%, and 2/6 respectively, which remain consistent with the previous left–right case.

In Fig. 9, after the excavation of up tunnel, the maximum principal stress of the surrounding rock of the tunnel is significantly reduced, which is mainly manifested in the sharp reduction of the normal stress and the increase of the tangential stress. After the excavation of twin tunnels, compared with the initial state, the maximum principal stress changes obviously, especially for the normal stress around the tunnel, which is approximately 0.

In Fig. 10, after the excavation of the up tunnel, the closer the distance to the arch waist of up tunnel, the denser the distribution of YAI contours, the smaller the YAI (reduces from 0.85 to 0.55), and the less safe it is. The contours of YAI near the up tunnel are distributed symmetrically, with distinct “ear-shaped” formations on both sides of the tunnel. Conversely, the contours beneath the bottom arch of the upper tunnel are sparsely distributed, resembling a “long-melon” shape.

After the twin tunnels excavation, the YAI contours near the two tunnels distributes symmetrically, and YAI contours on both left and right sides of rock pillar between the twin tunnels distributes in a double spiral. Among them, the arch on the two sides of the twin tunnels is the most dangerous part, since the YAI is the smallest (the value is 0.55). Therefore, for the excavation of the up-down twin tunnels, reinforce the lining and enhance monitoring of the arch waists on both sides of the twin tunnels should be strengthened to prevent yield failure.

#### Comparison and discussion of the stability of left–right twin tunnels and up-down twin tunnels

From the previous discussion, there are still many differences in the calculation results of these two tunnel layouts. After the excavation of single tunnel, the YAI of the up-down twin tunnels is much smaller than that of the left–right twin tunnels. After the excavation of the twin tunnels, the two sides’ waists of the up-down twin tunnels pose the highest risk while the rock pillar of the left–right twin tunnels is the most dangerous. The YAI value of the former is greater, so it is safer, which indicates that the arrangement of left–right twin tunnels is very unfavorable to the mechanical stability of the twin tunnels.

**Figure 11:**
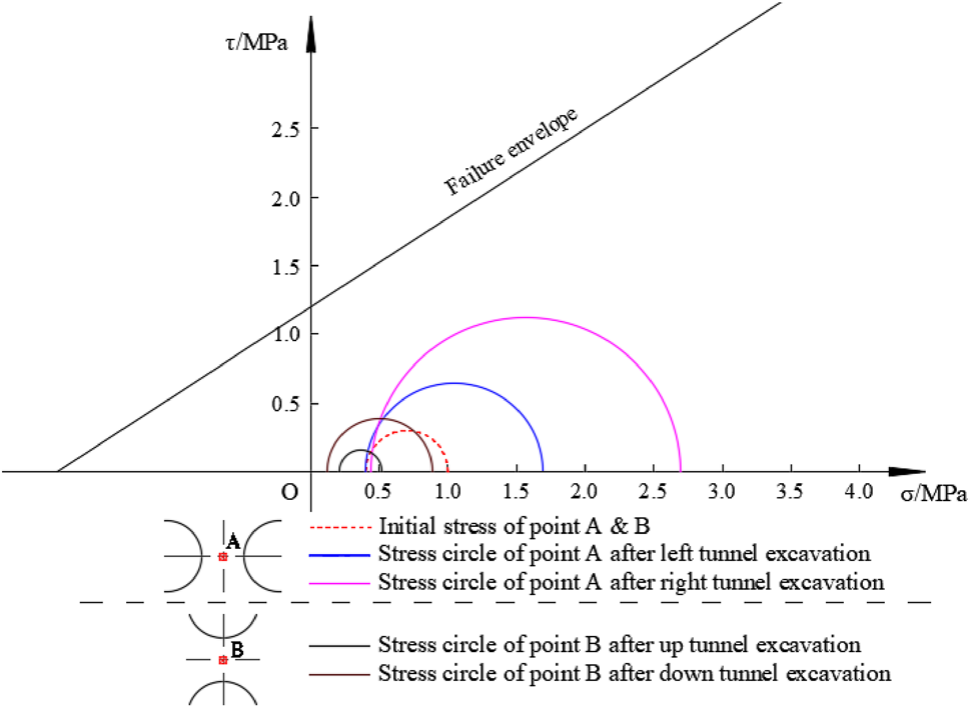
The comparison of Mohr’s circle of left–right twin tunnels and up-down twin tunnels.

Similarly, Fig. 11 presents a comparison of Mohr’s circle for the left–right and up-down twin tunnels based on the numerical results.

In Fig. 11, after the excavation of one tunnel or twin tunnels, the stress circle of up-down twin tunnels is much smaller than that of the left–right twin tunnels, and the former’s radius is about half of the latter and is less susceptible to yield failure. The arrangement of up-down twin tunnels is more reasonable, which is more conducive to the mechanical stability performance of twin tunnels. At the same time, the stress circle shrinks sharply after a single tunnel excavation for the case of up-down twin tunnels. This is because the excavation of tunnel, leading to the decreasing in σ_1_ and increasing in the σ_3_, and this two principle stress may even equal (i.e. σ_1_ = σ_3_) and the stress circle is just a point.

## Influencing factors analysis of the stability differences between left–right twin tunnels and up-down twin tunnels

The mechanical stabilities of two tunnels with small clearance are greatly affected by SPC, SRR, and RPT/TD, but the influence degree of each of these factors is not the same. Therefore, multiple sets of calculation conditions are designed in this section to further quantify the changes in the mechanical stability of the up-down tunnels and the left–right tunnels under various influencing factors, aiming to provide some important references for engineering design.

### Influence of SPC

**Figure 12:**
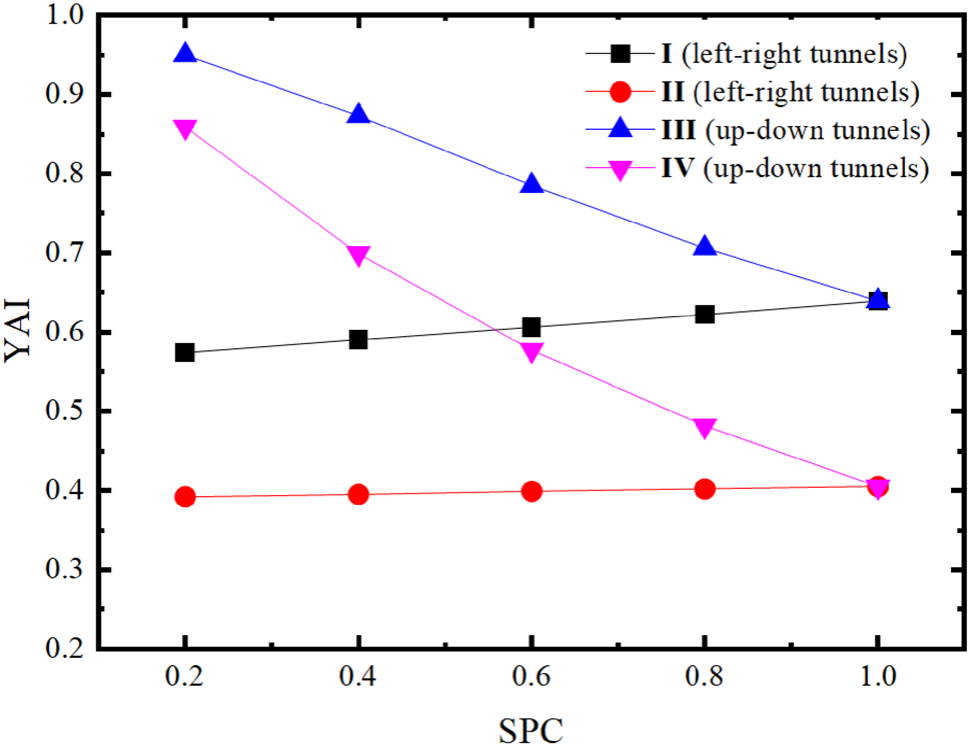
The comparison curve of SPC and YAI of up-down tunnels and left–right tunnels.

By exchanging the stress of the x and y direction, the stability changes of the rock pillar for twin tunnels under different SPC are simulated and analyzed. To control the single variable, the SRR and RPT are fixed at 100% and 2m, respectively. Five kinds of SPC for twin tunnel analysis are selected, as shown in Table 3. The calculated YAI of the middle point of rock pillar is shown in Table 4. Meanwhile, to show the changing law of YAI more vividly, the data in Table 4 is drawn into a curve, as shown in Fig. 12.

**Table 3:** Initial stress field of twin tunnels with different SPC.

SPC	Initial stress field	Left–right twin tunnels/MPa	Up-down twin tunnels/MPa
0.2	$\sigma_{x}$	0.2	1.0
	$\sigma_{y}$	1.0	0.2
0.4	$\sigma_{x}$	0.4	1.0
	$\sigma_{y}$	1.0	0.4
0.6	$\sigma_{x}$	0.6	1.0
	$\sigma_{y}$	1.0	0.6
0.8	$\sigma_{x}$	0.8	1.0
	$\sigma_{y}$	1.0	0.8
1.0	$\sigma_{x}$	1.0	
	$\sigma_{y}$	1.0

**Table 4:** YAI of the middle point of rock pillar under different SPC.

SPC	YAI			
	Left–right tunnels		Up-down tunnels	
	I	II	III	IV
0.2	0.574	0.392	0.950	0.859
0.4	0.590	0.395	0.873	0.699
0.6	0.606	0.399	0.785	0.577
0.8	0.622	0.402	0.706	0.482
1.0	0.639	0.405	0.639	0.405

I refer to “after excavation of the left tunnel”; II refers to “excavation about the left and right tunnels”; III refers to “after excavation of the up tunnel”; IV refers to “after excavation of the up and down tunnels”.

In Table 4 and Fig. 12, YAI is the largest after the excavation of a single tunnel under different SPC. As compared with twin tunnels, single tunnel presents greater stability under various SPC. As SPC increases from 0.2 to 1.0, YAI also increases from 0.392 to 0.405 in the case of the left–right twin tunnels, meaning that rock pillar gradually become safer. While for the case of up-down twin tunnels, YAI shows a decreasing trend (from 0.859 to 0.405) as the SPC increases from 0.2 to 1.0. After the excavation of two tunnels, the YAI of the up-down twin tunnels under different SPC is greater than that of the left–right twin tunnels. Compared with the left–right twin tunnels, the YAI change of the up-down twin tunnels is more obvious, since the curve is steeper. As SPC increases from 0.2 to 1, the YAI of the rock pillar of left–right twin tunnels increases by 3.3%, while that of the rock pillar of up-down twin tunnels decreases by 52.9%. It’s concluded that when compared with the left–right twin tunnels, the stabilities of up-down twin tunnels are more sensitive to SPC.

In Fig. 12, it is also worthwhile mentioning that the SPC-YAI function of the rock pillar between left–right twin tunnels appeared as a straight line since the change in the sum of $\sigma_{1}$ and $\sigma_{3}$(i.e. $\sigma_{1} + \sigma_{3}$) is not obvious with the increasing of SPC. This means that the denominator of YAI is approximately constant, while the gap between $\sigma_{1}$ and $\sigma_{3}$ (i.e. $\sigma_{1} - \sigma_{3}$) is generally expressed as a linear relationship of SPC. These factors make SPC show a linear relationship to YAI.

### Influence of SRR

**Figure 13:**
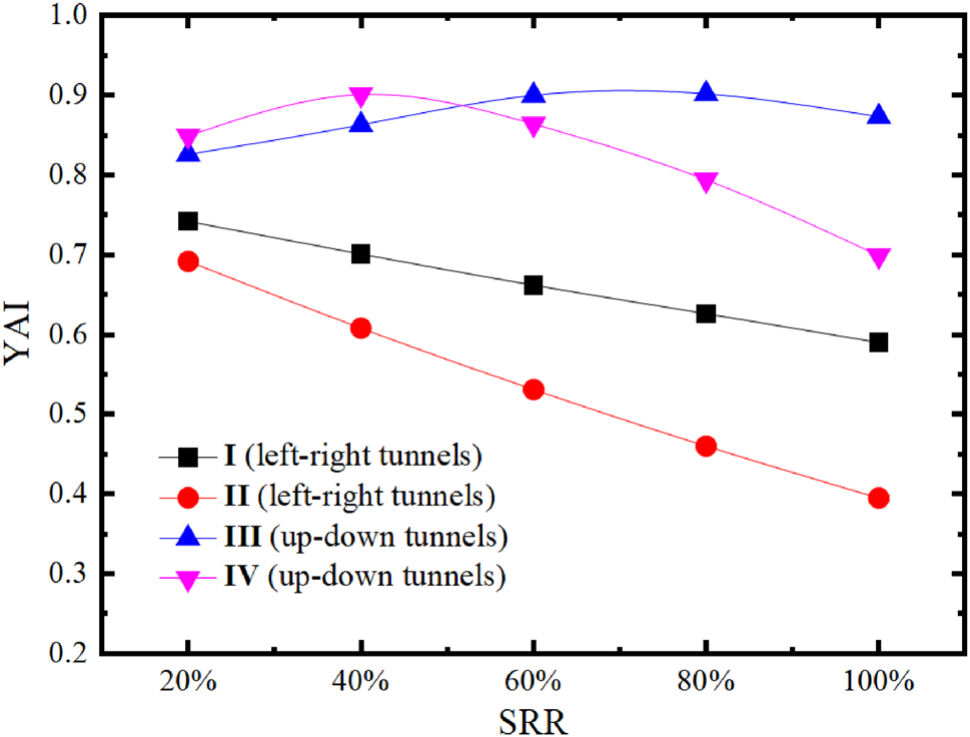
The comparison curve of SRR and YAI of up-down tunnels and the left–right tunnels.

Similarly, to control the single variable, SPC and RPT are fixed at 0.4 and 2 m, respectively. To further study the influence of SRR on the stability of rock pillar in the up-down tunnels and left–right tunnels, different SRRs (20%–100%) are selected for the numerical calculation model respectively, and the YAI of rock pillar is analyzed under different SRR, as shown in Table 5. The relationship curve between SRR and YAI is drawn according to Table 5, as shown in Fig. 13.

**Table 5:** YAI of middle point about rock pillar under different SRR.

SRR	YAI			
	Left–right tunnels		Up-down tunnels	
	I	II	III	IV
20%	0.742	0.692	0.826	0.849
40%	0.701	0.608	0.863	0.901
60%	0.662	0.531	0.900	0.864
80%	0.626	0.460	0.902	0.794
100%	0.590	0.395	0.873	0.699

In Fig. 13 and Table 5, the change law of YAI with different SRR is visual. The SRR reflects the impact of the excavation on the rock. The rock pillar between up-down twin tunnels exhibits superior stability performance than that between left–right twin tunnels. For the case of up-down twin tunnels, the function of SRR and YAI is not monotonic. This phenomenon results from the fact that tunnel excavation with lower SRR can be considered as an increase in the tangential stress and a decrease in the radial stress of the rock pillar. However, the directions of $\sigma_{1}$ and $\sigma_{3}$ do not change from the initial stress field. Therefore, in this case, $\sigma_{1}$ keeps decreasing and $\sigma_{3}$ keeps increasing with the increase of SRR, leading to the increase of YAI. This phenomenon will continue until the directions of $\sigma_{1}$ and $\sigma_{3}$ exchange. At this moment, the increase of SRR leads to the continuous increase of $\sigma_{1}$ and the decrease of $\sigma_{3}$, which results in the decrease of YAI.

**Figure 14:**
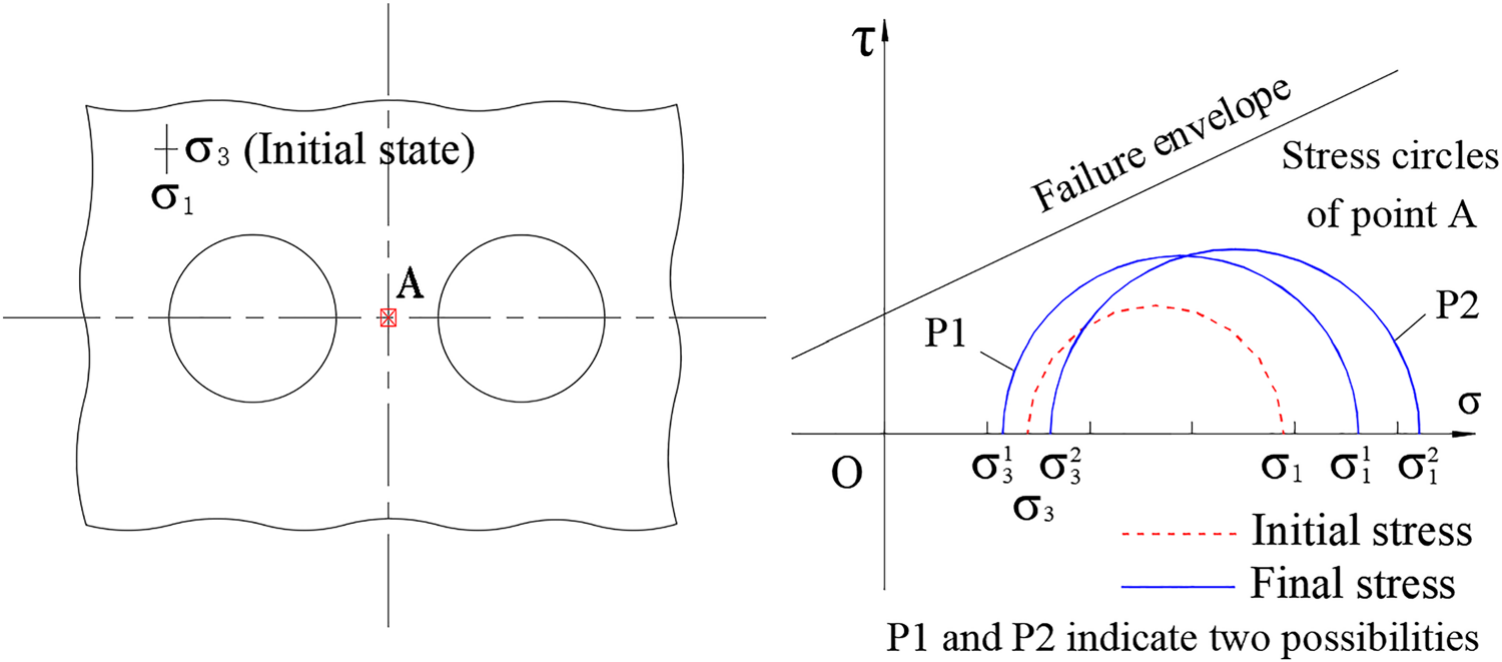
Schematic diagram of stresses evolution of point A (for left–right twin tunnels).

**Figure 15:**
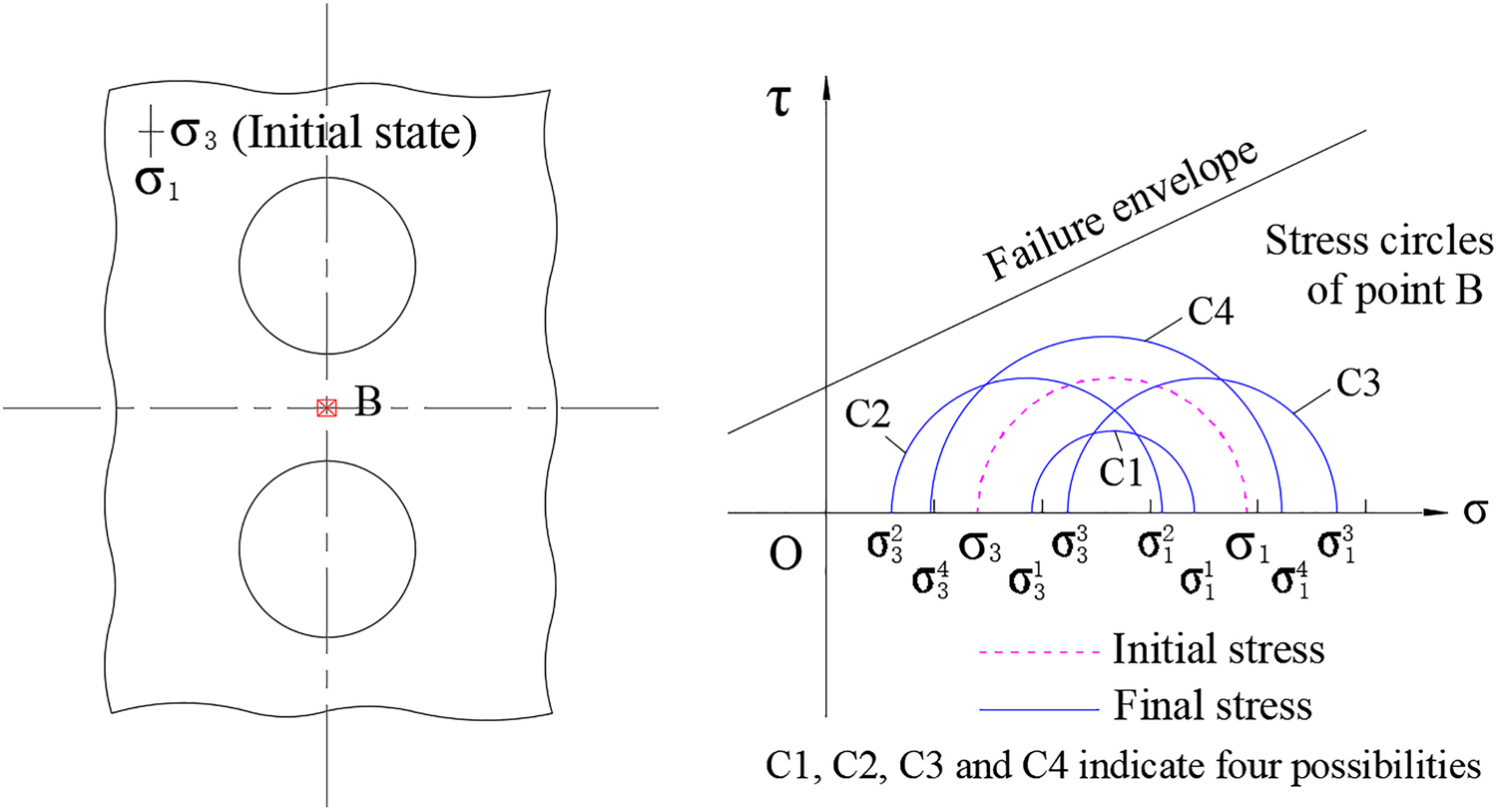
Schematic diagram of stresses evolution of point B (for up-down twin tunnels).

It should be noted that the YAI value have a turn point when the SRR is between 20% and 60% after the excavation of second tunnel of up-down tunnels while left–right tunnels case does not show similar turning point. The reasons are as follows: Excavation of tunnel will cause tangential stress concentration and radial stress release. Therefore, for the middle point (Point A in Fig. 14) in the rock pillar of left–right twin tunnels, assuming in the gravity stress field with maximum principal stress (σ_1_) vertical and minimum principal stress (σ_3_) transverse, the principal stress evolution can be qualitatively depicted as two cases shown in Fig. 14. Since both left and right tunnel excavations will cause tangential stress (in parallel to original σ_1_) increasing and radial stress (in parallel to original σ_3_) decreasing, the final principal stress can be illustrated as P1 in Fig. 14. However, the final elliptical deformations of twin tunnels, which would give Point A pressure at horizontal direction, may result in a bit increasing the σ_3_, as expressed by P2 in Fig. 14. Whether it is P1 or P2, one thing is clear that the principal stress direction is the same as original state. However, for the case of up-down twin tunnels, it is more complicated since tunnel excavations will cause original σ_1_ decreasing and original σ_3_ increasing. The final stress state depends on the degree of alteration, as shown in Fig. 15. If the original σ_1_ decreases and σ_3_ increases but still keeps the maximum principal stress vertical and minimum principal stress transverse, the Circle 1 (C1) will be this case. When the original σ_1_ decreases to be lower than σ_3_, namely the reverse happens with maximum principal stress transverse and minimum principal stress vertical, it comes as Circle 2, 3, 4 (C2, C3, C4) according to different degrees of alteration. Among these four different final states, some circles are closer to the failure envelope compared with the initial stress state, while some are farther off the failure envelope.

For up-down twin tunnels, under the premise that SRR is low and principal stress direction does not change, two tunnel excavations will result in more increase of $\sigma_{3}$ and decrease of $\sigma_{1}$ when compared to the single tunnel excavation, which leads the Mohr Circle to be farther off the failure envelope. It accounts for the counterintuitive finding that the excavation of two tunnels makes the rock pillar more stable than single tunnel excavation under a small SRR (in Fig. 13). Therefore, changing the construction method helps to reduce SRR, which can effectively improve the stabilities about the rock pillar of two tunnels.

### Influence of RPT/TD

**Figure 16:**
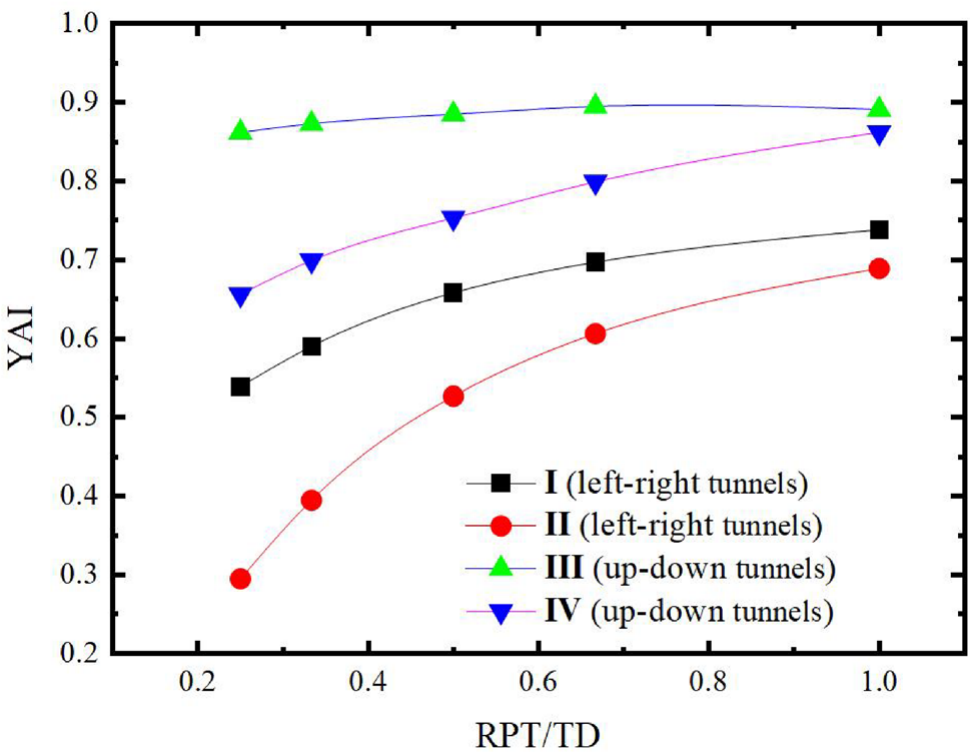
The relationship between RPT/TD and YAI.

Regarding the influence about RPT on the stability of twin tunnels, the common view is that the further distance between twin tunnels, the better stability of rock pillar will be. Through the evaluation of YAI, the impact of the change in RPT on the stabilities of rock pillar can be found more intuitively. The SPC and SRR are set to 0.4 and 100% respectively, and TD is fixed at 6m, while RPT/TD is changed from 1/4 to 1.0. Then, the YAI of the rock pillar are analyzed under different sets of RPT/TD for left–right twin tunnels and up-down twin tunnels, as shown in Table 6, and the relationship curve between YAI versus RPT/TD is shown in Fig. 16.

**Table 6:** Calculated YAI values of rock pillar under different RPT/TD.

RPT/TD	YAI			
	Left–right tunnels		Up-down tunnels	
	I	II	III	IV
1/4 (RPT = 1.5 m)	0.539	0.295	0.862	0.656
1/3 (RPT = 2 m)	0.590	0.395	0.873	0.699
1/2 (RPT = 3 m)	0.658	0.527	0.885	0.753
2/3 (RPT = 4 m)	0.697	0.606	0.895	0.799
1 (RPT = 6 m)	0.738	0.689	0.891	0.862

In Table 6 and Fig. 16, it can be found that the stabilities of rock pillar of up-down twin tunnels are better than the stabilities of left–right twin tunnels. The average slopes of these two curves are calculated from Fig. 16. For the case of left–right twin tunnels, the average slopes of the four-segment curves are 1.2, 0.797, 0.474, and 0.249 respectively. While for the case of up-down twin tunnels, it is changed to be 0.516, 0.323, 0.276, and 0.189. As RPT/TD continues to increase, YAI of rock pillar of left–right twin tunnels rises faster than that of the up-down twin tunnels. Therefore, it can be concluded that the left–right twin tunnels are more sensitive to RPT/TD.

Furthermore, the growth of YAI appears to be slower and slower with the continuous increase of RPT/TD. When RPT/TD is within 1/4 ~ 2/3, YAI enhances rapidly with RPT/TD. It demonstrates that increasing RPT between twin tunnels can effectively increase the stability of the rock pillar. However, it is no longer obvious to increase the stabilities of rock pillar when RPT increases to a certain extent. Also, whether it’s up-down twin tunnels or left–right twin tunnels, the YAI differences between the single tunnel and the twin tunnels is becoming very little with RPT/TD, especially when RPT /TD = 1.0 (i.e. the RPT is equal to TD).

## Conclusions

In this paper, the stability of rock pillar between twin tunnels with small clearance is investigated. Based on the analysis, the conclusions are as follows:

1. The numerical result is in good consistent with the theoretical result, verifying the rationality of numerical calculation. In the case of the left–right twin tunnels, the direction of the principal stresses (σ_1_ and σ_3_) remains consistent with the initial state throughout the excavation process. However, for the up-down twin tunnels, the principal stress direction varies significantly, and the final stress state is determined by the extent of alteration.
2. The YAI of up-down twin tunnels is significantly lower compared to that of left–right twin tunnels. The two side waists in the up-down twin tunnels and the rock pillar in the left–right twin tunnels pose the highest risk, and the arrangement of up-down twin tunnels provides better mechanical performance for the twin tunnel system.
3. The stability of rock pillar in up-down twin tunnels is primarily affected by SPC, whereas the stability of rock pillar in left–right twin tunnels is more sensitive to SRR. To enhance the stability of rock pillar, two approaches can be taken: reducing the SRR by modifying the construction method and increasing RPT/TD within the range of 1/4 to 2/3. It is important to note that certain ideal assumptions are made during the calculations, which are valid for favorable surrounding rock conditions. However, in the case of poor surrounding rock, the reduction coefficient can be used.

## Data Availability

Data will be available by the corresponding author on reasonable request.
